# Supplementary material for: Metabolomics combined with clinical analysis explores metabolic changes and potential serum metabolite biomarkers of antineutrophil cytoplasmic antibody-associated vasculitis with renal impairment
Source: PeerJ. 2023 Mar 15;11:e15051. doi: 10.7717/peerj.15051 (PMC10024486; doi:10.7717/peerj.15051)
Supplement: Supplemental Information 3 — The curves of total ion current of quality control samples were highly overlapped. (A) total ion current overlap pattern of quality control samples in negative ion mode; (B) total ion current overlap pattern of quality control samples in positive ion mode. [file peerj-11-15051-s003.pdf]

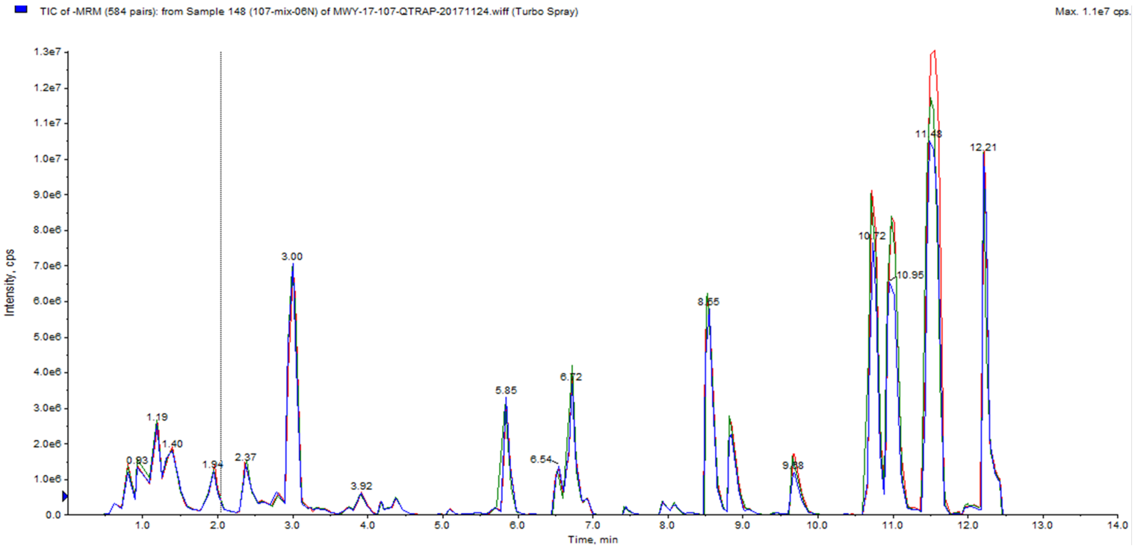

A

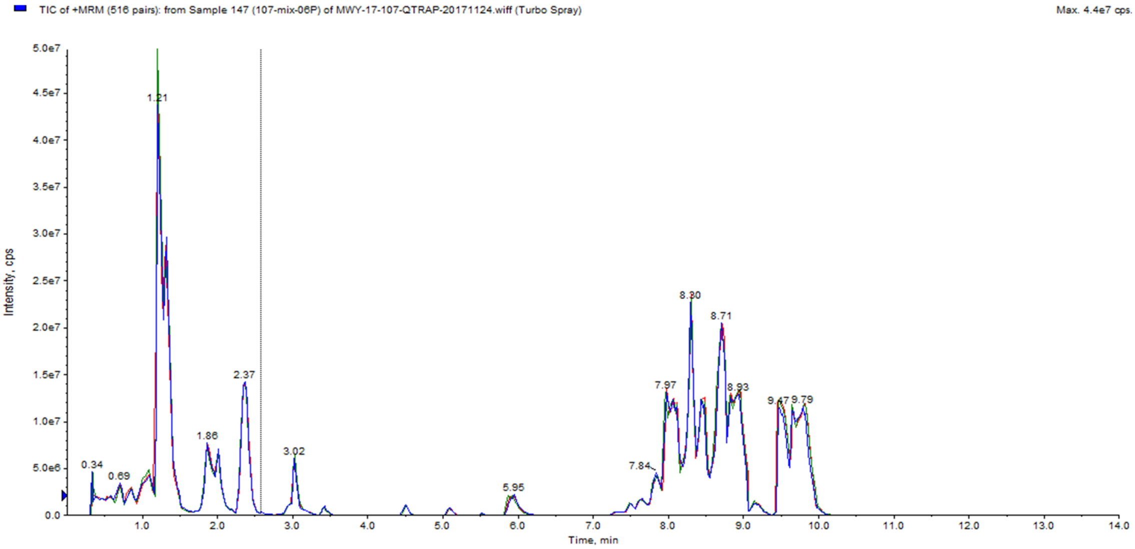

B

Fig. S1 The curves of total ion current of quality control samples were highly overlapped. (A) total ion current overlap pattern of quality control samples in negative ion mode; (B) total ion current overlap pattern of quality control samples in positive ion mode.
